# Supplementary material for: Wheat Metabolite Interferences on Fluorescent Pseudomonas Physiology Modify Wheat Metabolome through an Ecological Feedback
Source: Metabolites. 2022 Mar 9;12(3):236. doi: 10.3390/metabo12030236 (PMC8955329; doi:10.3390/metabo12030236)
Supplement: Supplementary file 1 [file metabolites-12-00236-s001.zip › Table S1.pdf]

Table S1. Parameter settings used for metabolomics data processing of *Pseudomonas* extract and wheat root samples

Step	Method	Parameters	Values wheat root samples	Values *Pseudomonas* strains culture samples
**Peak detection (*Peakpicking*)** [a]	centWave	ppm	10	10
		mzdiff	0.05	0.05
		prefilter	3, 2500	3, 5000
		snthresh	10	10
		peakwidth	4, 15	4, 15
		noise	5000	10000
**Peak grouping (*group*)** [a]	density	bw	4	3
		mzwid	0.25	0.25
		minfrac	0.05	0.01
**FillPeaks**	chrom			

[a] data processed with xcms R-package on collaborative Galaxy platform "Workflow4metabolomics" version 3.3
