# Supplementary material for: Wheat Metabolite Interferences on Fluorescent Pseudomonas Physiology Modify Wheat Metabolome through an Ecological Feedback
Source: Metabolites. 2022 Mar 9;12(3):236. doi: 10.3390/metabo12030236 (PMC8955329; doi:10.3390/metabo12030236)
Supplement: Supplementary file 1 [file metabolites-12-00236-s001.zip › Table S2.pdf]

Table S2. Molecular networking: MZmine 2 data-preprocessing parameters

Steps	Methods	Parameters	Values
**Mass detection**		Noise level MS1	0
		Noise level MS2	0
**chromatogram builder**	ADAP	Minimum group size of scan	4
		Group intensity threshold	3000
		Minimum highest intensity	4000
		m/z tolerance	0.005 (20 ppm)
**Deconvolution**	ADAP Wavelets algorithm	S/N threshold	8
		Minimum feature height	4000
		Coefficient/area threshold	20
		Peak duration range	0.05 – 1 min
		T_R wavelet range	0.01 – 0.07 min
**MS2 scans paired**		m/z tolerance	0.02 Da
		t_R tolerance	0.3 min
**Isotopologue grouping**	Isotopic peak grouper algorithm	m/z tolerance	0.005 (20 ppm)
		t_R tolerance	0.2 min
**Filtering**	Feature list rows filter	Retention time range	1 – 14 min
		**Keep only peaks with MS2 scan**	
**Peak alignment**	Join aligner module	m/z tolerance	0.005 (20 ppm)
		Weight for m/z	2
		t_R tolerance	0.5 min
		Weight for t_R	1
**Gap filled**		m/z tolerance	0.005 (20 ppm)
		t_R tolerance	0.5 min
