# Supplementary material for: Wheat Metabolite Interferences on Fluorescent Pseudomonas Physiology Modify Wheat Metabolome through an Ecological Feedback
Source: Metabolites. 2022 Mar 9;12(3):236. doi: 10.3390/metabo12030236 (PMC8955329; doi:10.3390/metabo12030236)
Supplement: Supplementary file 1 [file metabolites-12-00236-s001.zip › Table S3.pdf]

Table S3. List of 15 the chemical standards used in UHPLC-DAD-qTOF analyses

N°	Name	Molecular formula	Origin	Purity
1	Monoacetylphloroglucinol	$C_8H_8O_4$	Cayman Chemical	≥98%
2	2,4-diacetylphloroglucinol	$C_{10}H_{10}O_5$	Toronto Research Chemicals Inc.	≥98%
3	Pyrrolnitrin	$C_{10}H_6Cl_2N_2O_2$	SIGMA Chemical Co.	≥98%
4	Phenazine-1-carboxylic acid	$C_{13}H_8N_2O_2$	Toronto Research Chemicals Inc.	≥98%
5	3-OH-C6-HSL	$C_{10}H_{17}NO_4$	SIGMA Chemical Co.	≥98%
6	Dimethyl 2,6-pyridinedicarboxylate	$C_9H_9NO_4$	SIGMA Chemical Co.	≥98%
7	Indole-acetic-acid	$C_{10}H_9NO_2$	SIGMA Chemical Co.	≥98%
8	Tryptophan	$C_{11}H_{12}N_2O_2$	SIGMA Chemical Co.	≥98%
9	Phenylalanin	$C_9H_{11}NO_2$	SIGMA Chemical Co.	≥98%
10	Fumaric acid	$C_4H_4O_4$	SIGMA Chemical Co.	≥98%
12	Caffeic acid	$C_9H_8O_4$	SIGMA Chemical Co.	≥98%
13	Succinic acid	$C_4H_6O_4$	SIGMA Chemical Co.	≥98%
14	DIBOA-Glc	$C_{14}H_{17}NO_9$	-	-
15	MBOA	$C_8H_7NO_3$	SIGMA Chemical Co.	≥98%
