# Supplementary figures and images for: Wheat Metabolite Interferences on Fluorescent Pseudomonas Physiology Modify Wheat Metabolome through an Ecological Feedback
Source: Metabolites. 2022 Mar 9;12(3):236. doi: 10.3390/metabo12030236 (PMC8955329; doi:10.3390/metabo12030236)

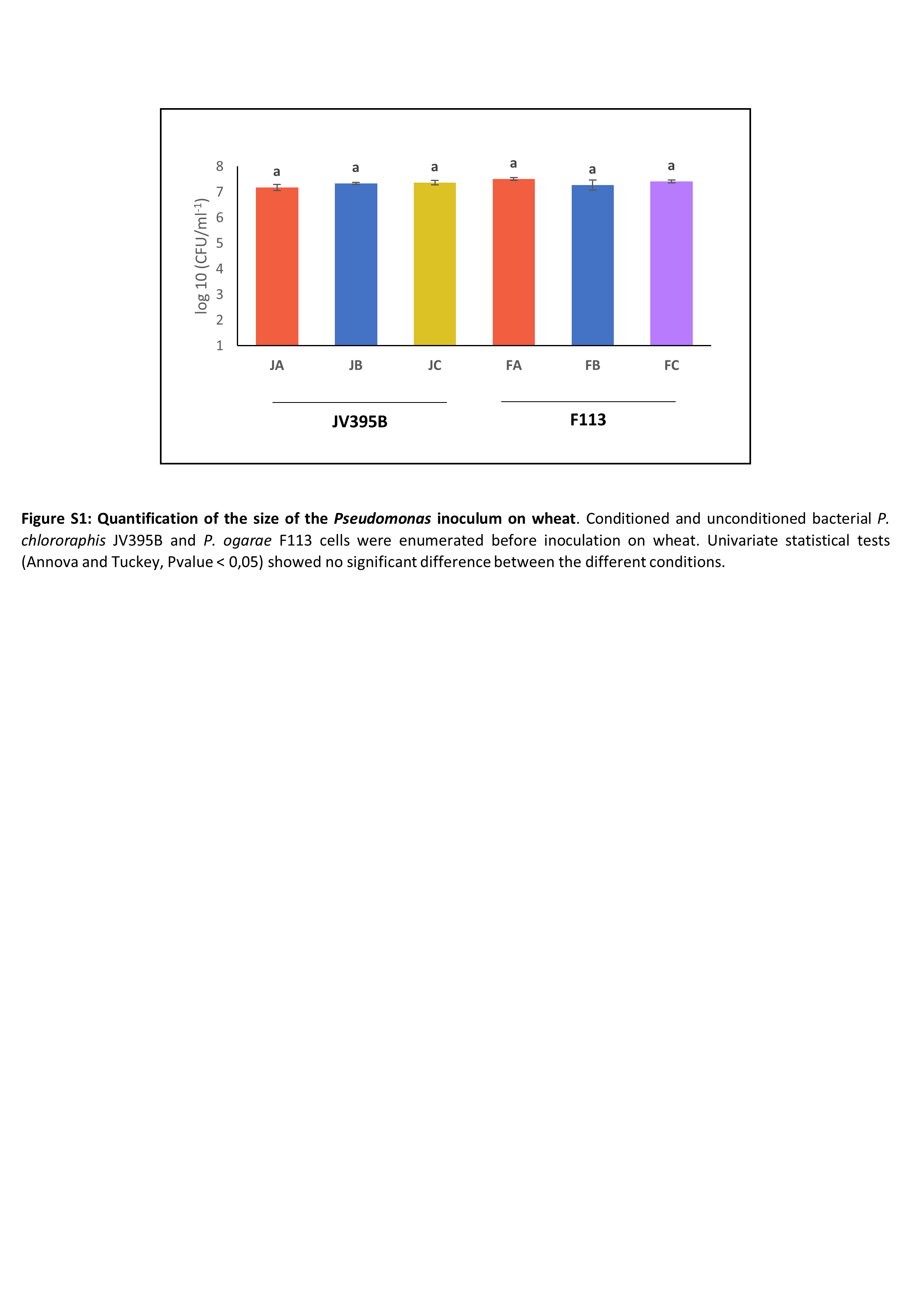

Supplement: Supplementary file 1 [file metabolites-12-00236-s001.zip › Figure S1.TIF]

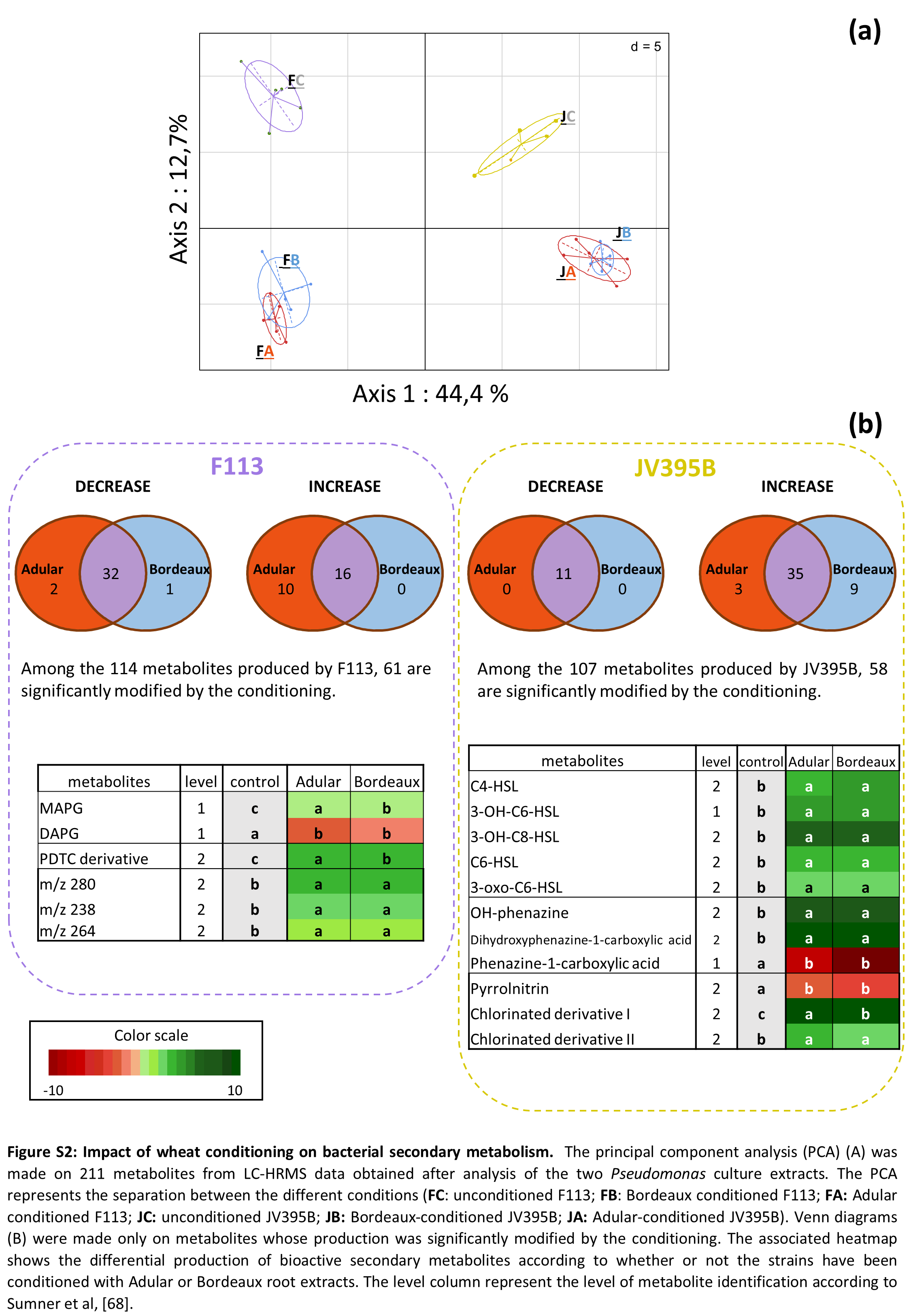

Supplement: Supplementary file 1 [file metabolites-12-00236-s001.zip › Figure S2.TIF]

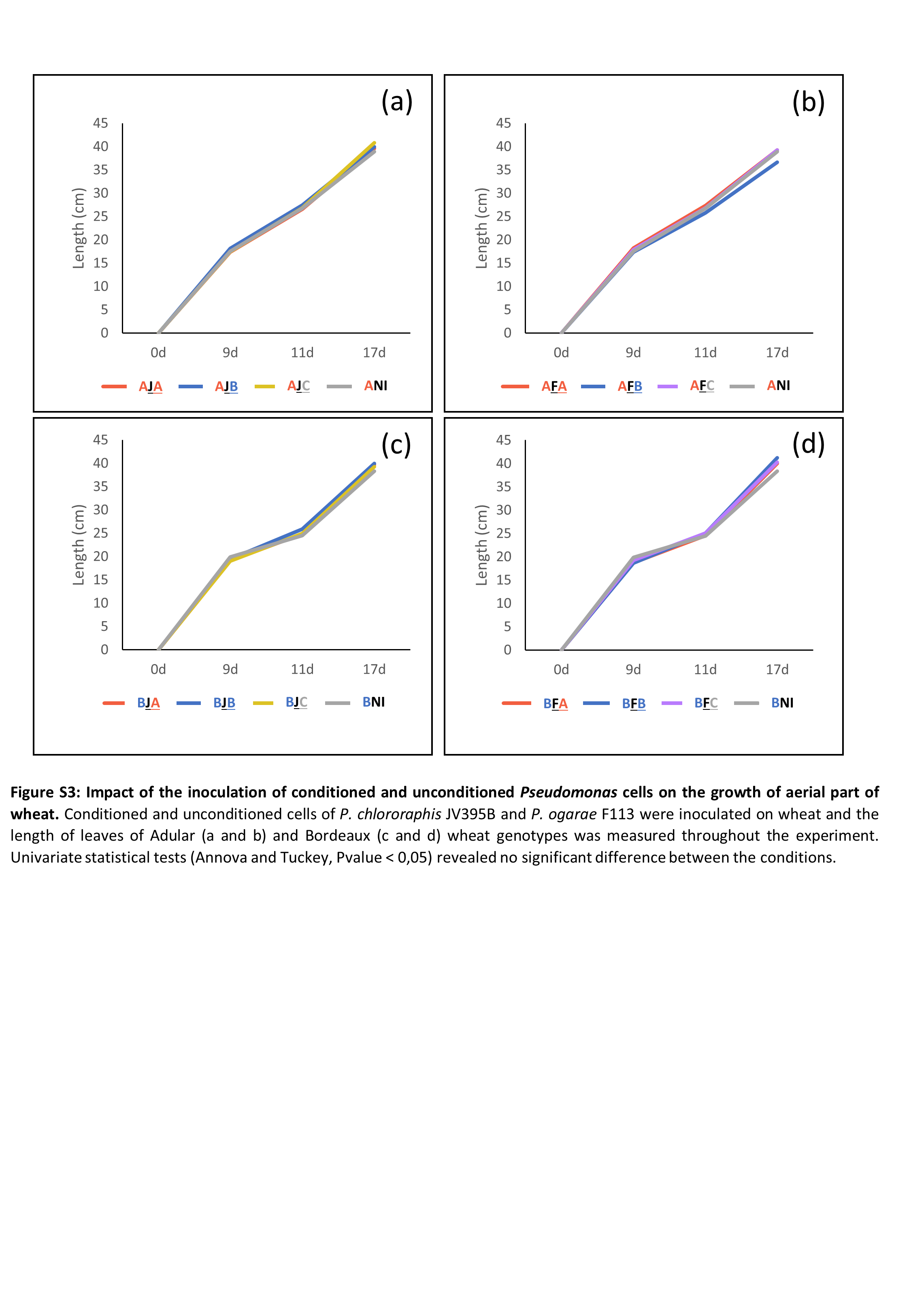

Supplement: Supplementary file 1 [file metabolites-12-00236-s001.zip › Figure S3.TIF]

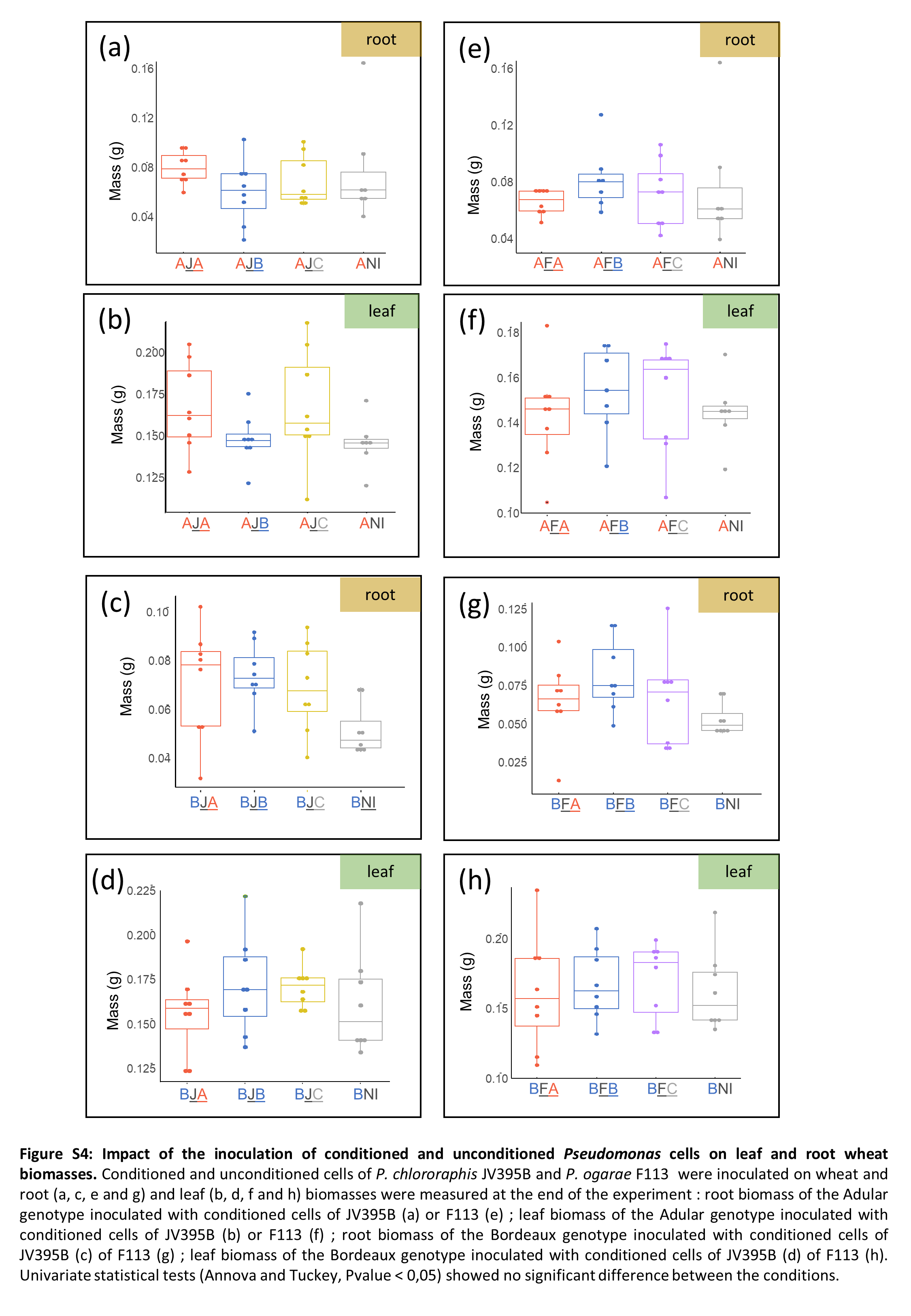

Supplement: Supplementary file 1 [file metabolites-12-00236-s001.zip › Figure S4.TIF]

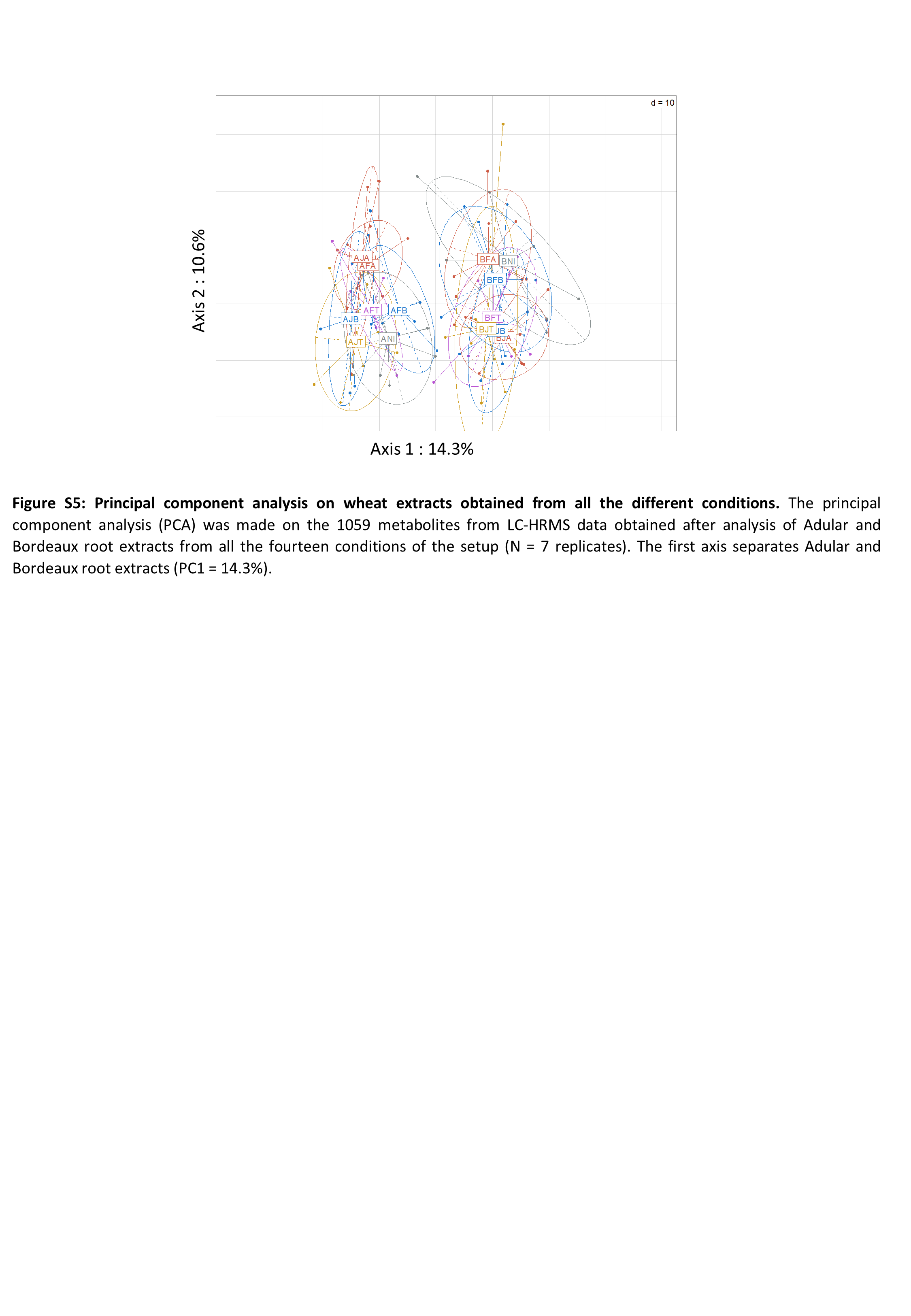

Supplement: Supplementary file 1 [file metabolites-12-00236-s001.zip › Figure S5.TIF]
